# Providers’ Experience with Artificial Intelligence-Based System During the COVID-19 Pandemic: Joe Lintz

**DOI:** 10.1093/eurpub/ckac131.001

**Published:** 2022-10-25

**Authors:** J Lintz

**Affiliations:** Health Information Management, Parker University, Dallas, Texas, USA

## Abstract

**Background:**

As the pandemic continues to spread worldwide, many of healthcare facilities are exploring new methods to keep their patients safe from potential hospital-acquired infections (HAIs) during the pandemic. This study explored the attitudes about artificial intelligence (AI) among providers who utilized AI-based hand hygiene monitoring system (HHMS) at a rural medical center during the pandemic.

**Methods:**

A self-administered questionnaire was mailed to 48 healthcare providers at a rural medical center in north Texas, with a 75% percent response rate (n = 36). The survey collected information on providers attitudes about AI-based HHMS use. In addition, the study also examined the relationship between provider’s well-being and the level of satisfaction with the AI-based HHMS use. The lessons learned from this study will be used to determine important factors to consider when attempting to advance and expand AI technologies in rural healthcare settings.

**Results:**

Results revealed that the integration of AI technology within the existing electronic health record (EHR) system remains a challenge for many providers. Plus, the lack of user-centered design approaches to incorporate the AI tool into existing workflows has reduced providers satisfaction about the new technology.

**Conclusions:**

The findings suggested that although AI technology has great promise to reduce the number of hospital-acquired infections (HAIs), successfully implement of an AI-based tool that meets the expectations of users requires significant levels of consolidation to ensure that it fits within the existing workflows and is accepted by users.

**Key messages:**

• AI application indirectly affects the well-being of providers, particularly in rural healthcare settings.

• A better AI application interface design that meets the expectations of providers is needed.

